# Pharmacists and the Insurance Bill

**Published:** 1911-07-15

**Authors:** 


### PHARMACISTS AND THE INSURANCE BILL.

A mass meeting of pharmacists was held in the King's Hall of the Holborn Restaurant, London, on Thursday last week for the purpose of considering proposed amendments of the National Insurance Bill, embodying the policy of the Council of the Pharmaceutical Society. About 900 pharmacists were present, the gathering being by far the largest of the kind that has ever been held in this country. A resolution expressing approval of the Council's action was carried, there being only three dissentients. The objects of the proposed amendments are (1) to secure that the dispensing of medicines under the proposed scheme shall be done by duly qualified pharmacists, and that no agreement for the supply of medicines shall be made except with those legally entitled to keep chemists' shops; (2) to provide that insured patients shall have free choice of chemist's shop; (3) to provide that remuneration shall be upon a scale system, the scale of prices to be fixed by local chemists and local Health Committees; (4) to place the control of medical and pharmaceutical services in the hands of the Health Committees; (5) to secure that pharmacy shall be represented on local Health Committees and on the Advisory Committee.

